# Increased Acquired Cholesteatoma Risk in Patients with Osteoporosis: A Retrospective Cohort Study

**DOI:** 10.1371/journal.pone.0132447

**Published:** 2015-07-14

**Authors:** Tang-Chuan Wang, Che-Chen Lin, Chia-Der Lin, Hsiung-Kwang Chung, Ching-Yuang Wang, Ming-Hsui Tsai, Chia-Hung Kao

**Affiliations:** 1 Department of Otolaryngology Head and Neck Surgery, China Medical University Hospital, Taichung, Taiwan; 2 Department of Otolaryngology Head and Neck Surgery, University of Iowa Hospital, Iowa City, IA, United States of America; 3 Management Office for Health Data, China Medical University Hospital, Taichung, Taiwan; 4 College of Medicine, China Medical University, Taichung, Taiwan; 5 Graduate Institute of Clinical Medical Science and School of Medicine, College of Medicine, China Medical University, Taichung, Taiwan; 6 Department of Nuclear Medicine and PET Center, China Medical University Hospital, Taichung, Taiwan; Georgia Regents University, UNITED STATES

## Abstract

**Objective:**

Clinically, we found the increased incidence of acquired colesteatoma in the patients with osteoporosis. In this study, we used a retrospective cohort to examine this association and to investigate the possible mechanism.

**Methods:**

We conducted a population-based retrospective cohort study by using the National Health Insurance Research Database (NHIRD). We identified an osteoporosis cohort comprising 37 124 patients newly diagnosed with osteoporosis aged 20 years or older. Patients in the comparison cohort had no history of osteoporosis and were frequency matched with the patients in the osteoporosis cohort according to sex, age, and index year.

**Results:**

The acquired cholesteatoma incidence rates for the osteoporosis and comparison cohorts were 1.12 and 0.83 per 1000 person-years, respectively. After we adjusted for confounding factors, the osteoporosis cohort exhibited a 1.32-fold increased acquired cholesteatoma risk relative to the comparison cohort (hazard ratio [HR] = 1.32, 95% confidence interval [CI] = 1.11–1.57). In addition, patients with no history of otitis media (HR = 1.33, 95% CI = 1.11–1.59), cancer (HR = 1.34, 95% CI = 1.12–1.60), or COPD (HR = 1.26, 95% CI = 1.05–1.52) in the osteoporosis cohort exhibited an increased risk of subsequent acquired cholesteatoma relative to those in the comparison cohort.

**Conclusions:**

Our cohort study indicated that patients with osteoporosis had a 1.31-fold increased acquired cholesteatoma risk relative to the comparison cohort. This risk was further increased in patients with comorbid otitis media. Hence, we recommend that otolaryngologists evaluate the condition of the middle ear of patients with osteoporosis.

## Introduction

Advances in healthcare and increases in life expectancy have caused osteoporosis and related fractures to become crucial health concerns worldwide, particularly among older adults [1]. The World Health Organization has emphasized the importance of osteoporosis. Osteoporosis-related fractures, which are prevalent in the older population, can lead to complications and even death. Osteoporosis is caused by reductions in bone mass and destruction of fine structures, which reduce the mechanical integrity of bone and increase the accumulation of noninvasive fractures [2].

Bone normally maintains an equilibrium balance of metabolic activity; however, when bone absorption occurs at a higher rate than bone production, bone volume remains unchanged, but bone gaps become larger and bone density decreases. Bone loss is progressive and no distinct symptoms appear at the onset. Osteoporosis is called the "silent disease" and is easily ignored. However, fractures and lower back pain attributable to osteoporosis are often key factors affecting the quality of life of older adults.

In addition to bone fracture, lower back pain, and other health-related consequences, previous studies have reported that osteoclast is associated with middle ear acquired cholesteatoma, the destructive expansion of a keratinizing squamous epithelium in the middle ear or petrous apex[3–5]. The mechanisms underlying the molecular and cellular pathogenesis of acquired middle ear acquired cholesteatoma are not fully understood [6]. Acquired cholesteatoma is not a malignant disease; however, the pathological process may lead to destruction of the surrounding bone, including the ossicles.

Little is known regarding the risk factors for cholesteatoma. It is generally accepted that cholesteatoma may be congenital or acquired, the latter occurring far more frequently. Even the pathogenesis of acquired cholesteatoma has been debated for many years, there are four basic theories of the pathogenesis of acquired aural cholesteatoma: (1)invagination of the tympanic membrane (retraction pocket cholesteatoma), (2)basal cell hyperplasia, (3) epithelial ingrowth through a perforation (the migration theory), and (4) squamous metaplasia of middle ear epithelium [7]. A study which included 45,980 patients revealed that children with persistent or refractory middle ear disease who need ventilation tubes were at increased risk of cholesteatoma besides of well known factors like otitis media, tympanic membrane perforation and Eustachain tube dysfunction [8]. A few studies have indicated that acquired cholesteatoma is associated with bone formation and absorption, but most of these studies are case reports, animal tests, or cytology studies [3–5]. A previous study indicated that acquired cholesteatoma is associated with anatomical abnormalities or other bone diseases such as osteoporosis [9]. However, no large-scale epidemiological study has been conducted to investigate this association. This study examined whether patients with osteoporosis may subsequently develop middle ear acquired cholesteatoma and whether other risk factors interact with osteoporosis to influence the development of acquired cholesteatoma.

## Materials and Method

### Data sources

The National Health Insurance Research Database (NHIRD), which was established in 1996, comprises data derived from the reimbursement claims of beneficiaries of the National Health Insurance (NHI) program, which covers more than 99% of the residents in Taiwan. The National Health Research Institutes (NHRI) maintains this database.

The study cohort was created using the Longitudinal Health Insurance Database (LHID), which is a subset of the NHIRD. The LHID was established from a randomly sampled set of one million people insured between 1996 and 2000. To protect the privacy of the insurants in the LHID, scrambled identification numbers were used to link the database before it was released for research use. In this study, diseases were classified according to the International Classification of Diseases, Ninth Revision, Clinical Modification (ICD-9-CM). The NHRID encrypts personal information to protect the privacy of the patients and provides researchers with anonymous identification numbers associated with relevant claims information, which includes the patients’ sex, date of birth, registry of medical services, and medication prescriptions.

### Study patients

This was a population-based retrospective cohort study. To create the case cohort, we identified patients aged 20 years or over who were newly diagnosed with osteoporosis (ICD-9-CM 733.0) from 1997 to 2008. The index date was the date of the osteoporosis diagnosis. The comparison cohort was composed of patients who had no history of osteoporosis and were frequency matched with the osteoporosis cohort according to sex, age, and index year. Patients with a history of acquired cholesteatoma (ICD-9 385.3) before the index date were excluded. The follow-up period ended when the development of acquired cholesteatoma was observed, when the patient withdrew from the insurance program, or at the end of 2009. Demographic factors and acquired cholesteatoma-associated comorbidities were listed as confounding factors. The examined comorbidities were cancer (ICD-9-CM 140–208 from catastrophic illness registry), chronic obstructive pulmonary disease (COPD, ICD-9-CM 250), otitis media (ICD-9-CM 381.0−381.4 and 382), hypertension (ICD-9-CM 401–405), diabetes mellitus (DM, ICD-9-CM 250), tympanic membrane perforation (TMP, ICD-9-CM 384) and eustachain tube dysfunction (ETD, ICD-9-CM 381) occurring before the index date. We also collected the osteoporosis patient’s bisphosphonate used information from index date to end of follow-up.

### Data Availability Statement

All data and related metadata were deposited in an appropriate public repository. The data on the study population that were obtained from the NHIRD (http://w3.nhri.org.tw/nhird//date_01.html) are maintained in the NHIRD (http://nhird.nhri.org.tw/). The NHRI is a nonprofit foundation established by the government.

### Ethics Statement

The NHIRD encrypts patient personal information to protect privacy and provides researchers with anonymous identification numbers associated with relevant claims information, including sex, date of birth, medical services received, and prescriptions. Patient consent is not required to access the NHIRD. This study was approved by the Institutional Review Board (IRB) of China Medical University (CMU-REC-101-012). The IRB specifically waived the consent requirement.

### Statistical analysis

We assessed the distribution of demographic factors and comorbidities of the osteoporosis and comparison cohorts, and the differences between the cohorts were tested using the chi-square test and *t* test. We calculated the acquired cholesteatoma incidence density based on newly diagnosed acquired cholesteatoma cases and person-years of follow up (from the index date to the acquired cholesteatoma incident or the end of follow-up) according to demographic status and comorbidity. The cumulative acquired cholesteatoma incidence curve of the 2 study cohorts was estimated using the product-limit method and the log-rank test. Cox proportional hazard regressions were used to estimate the hazard ratios (HRs) and 95% confidences intervals (CI) for the relative risk of acquired cholesteatoma. All analyses were performed using SAS statistical software (Version 9.3 for Windows; SAS Institute, Inc., Cary, NC, USA). The cumulative incidence curve was plotted using R software (R Foundation for Statistical Computing, Vienna, Austria). Values of *P* < .05 were considered statistically significant.

## Results

We established an osteoporosis cohort comprising 37 124 patients and a comparison cohort comprising 37 124 patients with similar average ages (63 y) and sex ratios (Table 1). Only 15% of osteoporosis patients was received the bisphosphonate treatment. Comorbidities were more prevalent in the osteoporosis cohort than in the comparison cohort (all *P* values < .0001).

**Table 1:** Baseline demographic characteristics and comorbidities between the Comparison and Osteoporosis groups.

Variable	Comparison group	Osteoporosis group	*P* value
	N = 37124 (%)	N = 37124 (%)	
**Age, mean (SD)** ^*^	63.2 (12.7)	63.3 (12.5)	0.3745
<50	5285 (14.2)	5285 (14.2)	>0.99
50–64	14173 (38.2)	14173 (38.2)	
65–74	9926 (26.7)	9926 (26.7)	
≥75	7740 (20.8)	7740 (20.8)	
**Gender**			>0.99
Female	30602 (82.4)	30602 (82.4)	
Male	6522 (17.6)	6522 (17.6)	
**Comorbidity**			
Otitis media	668 (1.8)	824 (2.2)	< 0.0001
TMP	58 (0.2)	89 (0.2)	0.0105
ETD	449 (1.2)	598 (1.6)	<0.0001
Cancer	1123 (3.0)	1340 (3.6)	<0.0001
COPD	4714 (12.7)	6662 (17.9)	<0.0001
Hypertension	16239 (43.7)	18667 (50.3)	<0.0001
DM	5521 (14.9)	6513 (17.5)	<0.0001
Bisphosphonate	-	5594 (15.1)	

* t-test

Abbreviation: COPD: chronic obstructive pulmonary disease; DM: Diabetes mellitus; TMP: tympanic membrane perforation; ETD: Eustachain tube dysfunction

**Fig 1:**
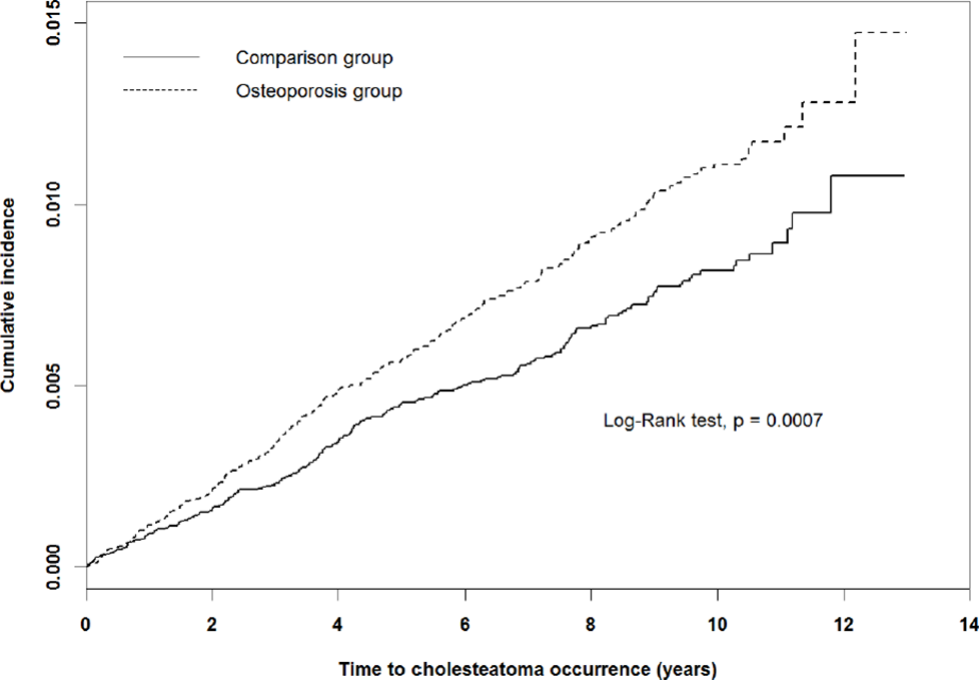
Cummulative incidence of acquired cholesteatoma for patients with (dashed line) or without (solid line) osteoporosis.

The subsequent acquired cholesteatoma incidence rates for the osteoporosis and comparison cohorts were 1.12 and 0.83 per 1000 person-years, respectively (Table 2). Fig 1 shows that the incidence curve for the osteoporosis cohort was significantly higher than for the comparison group (log-rank test *P* < .0001). After we adjusted for confounding factors, the osteoporosis cohort exhibited a 1.29-fold increased subsequent risk of acquired cholesteatoma relative to the comparison cohort (HR = 1.29, 95% CI = 1.09–1.54). The osteoporosis cohort was again associated with an increased risk of subsequent acquired cholesteatoma compared with the comparison cohort in patient aged 50–64 years (HR = 1.38, 95% CI = 1.01–1.89) and aged 65–74 years (HR = 1.42, 95% CI = 1.02–1.97). A sex-stratified analysis indicated that the risk of subsequent acquired cholesteatoma was increased relative to that of the comparison cohort among women (HR = 1.24, 95% CI = 1.03–1.50) and men (HR = 1.73, 95% CI = 1.04–2.86) in the osteoporosis cohort.

**Table 2:** Incidence rates and hazard ratios of developing acquired cholesteatoma.

	Comparison group			Osteoporosis group			Crude HR	Adjusted HR†
Variable	Case	PYs	Rate	Case	PYs	Rate	(95% CI)	(95% CI)
**Overall**	220	265429	0.83	306	273882	1.12	1.35(1.13–1.60)	1.29(1.09–1.54)
**Age group**								
<50	40	44012	0.91	51	44189	1.15	1.27(0.84–1.92)	1.24(0.85–1.80)
50–64	94	115651	0.81	148	116793	1.27	1.56(1.21–2.02)	1.38(1.01–1.89)
65–74	55	67897	0.81	74	70847	1.04	1.29(0.91–1.83)	1.42(1.02–1.97)
≥75	31	37869	0.82	33	42053	0.78	0.96(0.59–1.56)	1.09(0.73–1.63)
**Gender**								
Female	196	227810	0.86	263	236135	1.11	1.30(1.08–1.56)	1.24(1.03–1.50)
Male	24	37619	0.64	43	37747	1.14	1.79(1.09–2.95)	1.73(1.04–2.86)
**Comorbidity**								
**Otitis media**								
No	205	261680	0.78	282	268924	1.05	1.34(1.12–1.60)	1.30(1.08–1.56)
Yes	15	3749	4.00	24	4958	4.84	1.21(0.63–2.30)	1.17(0.61–2.25)
**TMP**								
No	218	265145	0.82	302	273427	1.10	1.34(1.13–1.60)	1.29(1.09–1.54)
Yes	2	284	7.03	4	455	8.79	1.23(0.22–6.71)	2.16(0.29–16.1)
**Cancer**								
No	212	259856	0.82	295	265829	1.11	1.36(1.14–1.62)	1.31(1.10–1.57)
Yes	8	5574	1.44	11	8053	1.37	0.95(0.38–2.37)	0.96(0.37–2.46)
**COPD**								
No	202	241168	0.84	253	235093	1.08	1.29(1.07–1.55)	1.24(1.03–1.49)
Yes	18	24261	0.74	53	38790	1.37	1.86(1.09–3.18)	1.86(1.09–3.19)
**Hypertension**								
No	119	160791	0.74	147	145340	1.01	1.37(1.07–1.74)	1.33(1.05–1.70)
Yes	101	104638	0.97	159	128542	1.24	1.28(1.00–1.65)	1.26(0.98–1.62)
**DM**								
No	196	232334	0.84	252	230261	1.09	1.30(1.08–1.57)	1.25(1.04–1.51)
Yes	24	33096	0.73	54	43622	1.24	1.72(1.06–2.78)	1.67(1.03–2.71)
**ETD**								
No	213	263098	0.81	297	270331	1.10	1.36(1.14–1.62)	1.31(1.10–1.56)
Yes	7	2331	3.00	9	3551	2.53	0.84(0.31–2.26)	0.83(0.30–2.26)

PYs, person-years; Rate, incidence rate per 1000 person-years.

†Model adjusted for age, sex, otitis media, cancer and COPD.

Abbreviation: COPD: chronic obstructive pulmonary disease; DM: Diabetes mellitus; TMP: tympanic membrane perforation; ETD: Eustachain tube dysfunction.

Table 2 shows the results of a comorbidity-stratified analysis of subsequent acquired cholesteatoma risk. Patients who were not diagnosed with otitis media (HR = 1.30, 95% CI = 1.08–1.56), TMP (HR = 1.29, 95% CI = 1.09–1.54), cancer (HR = 1.31, 95% CI = 1.10–1.57), hypertension (HR = 1.33, 95% CI = 1.05–1.70) or ETD (HR = 1.31, 95% CI = 1.10–1.56) in the osteoporosis cohort exhibited a significant increased risk of subsequent acquired cholesteatoma relative to those in the control cohort. We also observed the cholesteatoma risk was increased in the osteoporosis patient without COPD (HR = 1.24, 95% CI = 1.03–1.49) or with COPD (HR = 1.86, 95% CI = 1.09–3.19), and patient without DM (HR = 1.25, 95% CI = 1.04–1.51) or with DM (HR = 1.67, 95% CI = 1.03–2.71).

Table 3 shows the effects of osteoporosis and comorbidities on the risk of acquired cholesteatoma development. The results suggested that osteoporosis and comorbidities jointly affected the subsequent development of acquired cholesteatoma, but no interaction between osteoporosis and comorbidities occurred (*P* > .05 for all interaction tests).

**Table 3:** Joint effect osteoporosis and comorbidities on acquired cholesteatoma.

Variable		Case	Rate	Adjusted HR (95% CI)
**Osteoporosis**	**Otitis media**			
No	No	205	0.78	ref
No	Yes	15	4.00	5.25(3.10–8.88)
Yes	No	282	1.05	1.34(1.12–1.61)
Yes	Yes	24	4.84	6.24(4.09–9.53)
**Osteoporosis**	**Cancer**			
No	No	212	0.82	ref
No	Yes	8	1.44	1.82(0.90–3.70)
Yes	No	295	1.11	1.37(1.15–1.63)
Yes	Yes	11	1.37	1.69(0.92–3.09)
**Osteoporosis**	**COPD**			
No	No	202	0.84	ref
No	Yes	18	0.74	0.96(0.59–1.56)
Yes	No	253	1.08	1.29(1.07–1.55)
Yes	Yes	53	1.37	1.75(1.29–2.39)
**Osteoporosis**	**Hypertension**			
No	No	119	0.74	ref
No	Yes	101	0.97	1.47(1.12–1.94)
Yes	No	147	1.01	1.36(1.07–1.73)
Yes	Yes	159	1.24	1.89(1.48–2.43)
**Osteoporosis**	**DM**			
No	No	196	0.84	ref
No	Yes	24	0.73	0.9(0.58–1.37)
Yes	No	252	1.09	1.3(1.08–1.57)
Yes	Yes	54	1.24	1.53(1.13–2.07)
**Osteoporosis**	**TMP**			
No	No	218	0.82	ref
No	Yes	2	7.03	8.68(2.16–34.96)
Yes	No	302	1.10	1.35(1.13–1.61)
Yes	Yes	4	8.79	10.6(3.94–28.5)
**Osteoporosis**	**ETD**			
No	No	213	0.81	ref
No	Yes	7	3.00	3.73(1.76–7.92)
Yes	No	297	1.10	1.36(1.14–1.63)
Yes	Yes	9	2.53	3.14(1.61–6.11)

Rate: incidence rate per 1000 person-years.

Model adjusted for age and sex.

*P* > 0.05 for all interaction tests.

Abbreviation: COPD: chronic obstructive pulmonary disease; DM: Diabetes mellitus; TMP: tympanic membrane perforation; ETD: Eustachain tube dysfunction.

## Discussion

Acquired cholesteatoma can occur in the meninges, central nervous system, skull bones, and, most commonly, the middle ear and mastoid region. We focused on acquired cholesteatoma of the middle ear and mastoid region, which may lead to the destruction of middle and inner ear structures, hearing loss, vestibular dysfunction, and facial paralysis as well as lethal intracranial complications [10].

The primary strength of this study lies in the large number of patients and use of a national healthcare database. Taiwan launched the NHI program in 1995 and it is operated by a single buyer, the government. All insurance claims are scrutinized by medical reimbursement specialists and undergo peer review. The diagnoses of acquired cholesteatoma and osteoporosis were based on ICD-9 codes determined by qualified clinical physicians during strict audits in the reimbursement process. Therefore, the diagnoses of acquired cholesteatoma and osteoporosis are accurate and reliable even if they were diagnosed by different doctors.

Our cohort study indicated that patients with osteoporosis exhibited a 1.31-fold increased risk of developing acquired cholesteatoma relative to the comparison cohort. The risk of developing acquired cholesteatoma was particularly increased in patients diagnosed with both osteoporosis and otitis media. Various factors related to inflammation and local pressure influence osteoclast-mediated bone resorption in pathologic conditions. Protein products released by acquired cholesteatoma, such as interleukins (IL-1α,-1β, and IL-6), tumor necrosis factor α, interferon β, and parathyroid-hormone-related protein, have been identified [11,12]. Three factors are involved in the process of bone resorption, namely (1) mechanical factors, which are related to pressure generated by the expansion of a acquired cholesteatoma as it accumulates increasing amounts of keratin and purulent debris [13–15]; (2) biochemical factors, which are due to bacterial elements (endotoxins), products of host granulation tissue (collagenase, acid hydrolases), and substances related to acquired cholesteatoma (growth factors, cytokines) [16–24]; and (3) cellular factors, which are predominantly induced by osteoclastic activity [3–5].

Bone morphogenesis and remodeling involve the synthesis of bone matrices by osteoclasts [25]. Bone resorption under physiological conditions represents a balance of local osteoblast and osteoclast activity [26]. In 2003, Hamzei indicated that the number of osteoclast precursor cells is markedly increased in the perimatrix of acquired cholesteatoma tissue [4]. These results indicated that inflammation related to acquired cholesteatoma induces bone resorption through the release of the osteoprotegerin ligand from activated T cells, triggering osteoclastogenesis.

In our study, after we adjusted for confounding factors, the osteoporosis cohort exhibited a 1.31-fold increased risk of developing acquired cholesteatoma compared with the comparison cohort. Moreover, we observed a 6.24-fold increased risk of developing acquired cholesteatoma in patients with osteoporosis and otitis media. Our results are similar to those reported by previous studies [4, 11–15]. This is the population-based epidemiologic study with such a large sample size.Although smoking may be associated with inflammation, we were unable to obtain information regarding the effect of smoking or drinking on the risk of acquired cholesteatoma [27].

A recent study demonstrated an association between treatment with bisphosphonates and the occurrence of cholesteatoma in osteoporosis [28]. We try to collect the history of bisphosphonates used in osteoporosis patients but observed that only a few osteoporosis patients received bisphosphonates treatment. In fact, bisphosphonates were not paid by our insurance before osteoporosis fractures happened. I guess the degree of osteoporosis of most of these patients in our study is mild and without fractures. Therefore, we found a few osteoporosis patients who received bisphosphonate treatment in the new analyses. However, the data for individual patient’s BMD (the severity of osteoporosis) is not available in the NHIRD. In addition, the data for the other anti-osteoporosis drugs such as vitamin D and calcium tablets were not included in the NHIRD, because the patients might buy these drugs in drugstores and paid by themselves. Hence, this study could not provide good answer to this question. Thus, more large-scale epidemiological studies should be conducted to investigate this question.

We concluded that the acquired cholesteatoma risk in patients with osteoporosis is increased. We recommend that future studies involve monitoring inflammatory mediators and otitis-media-related values in patients with osteoporosis as well as the incidence of acquired cholesteatoma. A significantly increased risk of developing acquired cholesteatoma was identified in patients with osteoporosis and otitis media in this study. Clinicians should pay attention to both osteoporosis-related injuries and middle ear symptoms in patients with osteoporosis. Acquired cholesteatoma screening should be included in health assessments of patients with osteoporosis. The primary treatment for patients with acquired cholesteatoma is surgical excision; however, certain patients may experience a recurrence after surgical excision. This type of treatment may provide a favorable alternative to surgical treatment.
